# SNP-Based Chromosomal Microarray Analysis for Detecting DNA Copy Number Variations in Fetuses with a Thickened Nuchal Fold

**DOI:** 10.17691/stm2021.13.6.08

**Published:** 2021-12-28

**Authors:** J.K. Kievskaya, N.V. Shilova, I.V. Kanivets, E.V. Kudryavtseva, D.V. Pyankov, S.A. Korostelev

**Affiliations:** Geneticist; Genomed Ltd., 8, Bldg 5 Podolskoe Shosse, Moscow, 115093, Russia;; Head of the Laboratory of Molecular Cytogenetics; Research Centre for Medical Genetics, 1 Moskvorechye St., Moscow, 115522, Russia;; Geneticist; Genomed Ltd., 8, Bldg 5 Podolskoe Shosse, Moscow, 115093, Russia; Associate Professor, Department of Medical Genetics; Russian Medical Academy of Continuous Professional Education, 2/1, Bldg 1 Barricadnaya St., Moscow, 125993, Russia;; Associate Professor, Department of Obstetrics and Gynecology; Ural State Medical University, 3 Repina St., Ekaterinburg, 620028, Russia; Head of the Laboratory, Geneticist; Genomed Ltd., 8, Bldg 5 Podolskoe Shosse, Moscow, 115093, Russia;; Professor, General Director; Genomed Ltd., 8, Bldg 5 Podolskoe Shosse, Moscow, 115093, Russia;

**Keywords:** chromosomal microarray analysis, prenatal diagnostics, nuchal fold thickness, DNA copies number variations, reciprocal translocation

## Abstract

**The aim of the study** was to assess the diagnostic potential of SNP-based chromosomal microarray analysis for detecting pathogenic copies number variations (CNVs) in fetuses with a normal karyotype, in which an increase in the nuchal translucence of >2.5 mm was detected by ultrasound at a gestational age of 11 weeks to 13 weeks 6 days.

**Materials and Methods:**

The study included 225 pregnant women who underwent invasive prenatal diagnostic procedures following the detection of an isolated thickening of the fetal nuchal fold. The fetal material obtained was examined using a cytogenetic test; if a normal karyotype was confirmed, chromosomal microarray analysis was performed as a second-line test.

**Results:**

Pathogenic CNVs were detected in 22 of 225 fetuses (9.8%) with a normal karyotype. Of these 22 fetuses, pathogenic CNVs not classified as syndromes were detected in 14 cases (63.6%), and those previously described as syndromes — in 8 cases (36.4%). In 9 fetuses (41%), CNVs in two non-homologous chromosomes were determined; these findings indicated a high likelihood of carrying balanced translocations in the parents. Indeed, when analyzing the parent’s karyotype, in 8 out of 9 couples, balanced translocations were found in one of the parents.

**Conclusion:**

Using chromosomal microarray analysis in fetuses with a thickened nuchal fold makes it possible to increase the ability to detect chromosomal imbalances, including those caused by pathological meiotic segregation of parental reciprocal translocation.

## Introduction

The most important and diagnostically significant ultrasound marker determined in the first trimester of pregnancy is the nuchal translucence (NT) [1]. The nuchal fold is the area between the inner surface of the fetal skin and the outer surface of the soft tissues that cover the cervical spine [2]. The normal values of NT, which is the ultrasound parameter of the nuchal fold thickness, depending on the gestational age and the coccygeal-parietal size of the fetus (Table 1) [2].

**Table 1:** Normal values of the nuchal fold thickness (mm) according to the gestational age

Gestational age	5^th^ percentile	50^th^ percentile	95^th^ percentile
11 weeks–11 weeks 6 days	0.9	1.3	2.1
12 weeks–12 weeks 6 days	1.0	1.6	2.4
13 weeks–13 weeks 6 days	1.2	1.8	2.5

With an increase in NT up to >2.5 mm, further karyotyping reveals chromosomal aneuploidies (CA) in about 38% of fetuses, and 61% of them are due to trisomy 21 [3, 4]. It is known that a thickened nuchal fold is a polypotential factor, since in these fetuses, not only CA frequency but also risks of congenital malformations (primarily heart defects) increase [5]. On average, 15% of fetuses with a normal karyotype and increased NT in the first trimester, may develop congenital malformations [4, 6]. A case, when the NT is increased, and the fetal chromosome set is normal, may indicate an increased risk of pregnancy complications at later stages [7, 8]; therefore, it is highly important to establish the cause that led to the appearance of an increased NT.

A thickening of the fetal nuchal fold may be associated not only with CA (such as trisomies 21, 18, and 13, etc.) but also with microdeletion/microduplication syndromes [9, 10]. These syndromes cannot be identified by a standard cytogenetic examination because the resolution of less than 8–10 million bp (nucleotide pairs) is needed to detect these changes [11]. The use of SNP-based chromosomal microarray analysis (CMA) for detecting genomic/chromosomal imbalances is advantageous compared with other methods not only in postnatal [12] but also in prenatal diagnostics [4]. CMA allows for diagnosing pathogenic variations in the number of DNA copies (copy number variations, CNV) in 5.6% of fetuses with isolated defects and in 9.1% with multiple developmental anomalies [13].

In prenatal diagnostics, the use of CMA was found to be more helpful than the karyotype analysis [4, 6]; thus, CMA is preferable to identify a chromosomal imbalance in the presence of fetal malformations detected by ultrasound. In 2012, a multicenter study organized by the National Institute of Child Health and Human Development (NICHD) showed that in fetuses with a normal karyotype and ultrasound-confirmed abnormalities, CMA was able to detect clinically significant CNVs in 6% of fetuses, and in the absence of ultrasound-confirmed findings — in 1.7% of fetuses [14]. Clinical guidelines from the American College of Obstetricians and Gynecologists (ACOG) and the Society for Maternal-Fetal Medicine (SMFM) underscore the importance of using CMA as a first-line test in cases of ultrasound-confirmed fetal abnormalities, while the traditional karyotype analysis is not required (level of evidence — 1A) [15].

It should be noted that the detection of clinically significant CNVs is possible by performing NGS (next-generation sequencing) tests. Today, NGS is the method of choice for detecting point mutations; however, even in this case, CMA remains the preferred method, since using NGS is associated with low concordance of CNV detection as found by all algorithms based on the number of reads. Compared to CMA, the NGS technique showed lower sensitivity and specificity in detecting clinically significant CNVs, and therefore CMA continues to play a key role in this methodology [16].

**The aim of the study** was to assess the diagnostic potential of chromosomal microarray analysis for detecting pathogenic CNVs in fetuses with a normal karyotype, in which an increase in the nuchal translucence of >2.5 mm was detected by ultrasound at a gestational age of 11 weeks to 13 weeks 6 days.

## Materials and Methods

This retrospective, cohort, descriptive study included 225 pregnant women aged 32 (20–44) years who underwent invasive prenatal diagnostic procedures in 2013–2020 following the detection of an isolated thickening of the nuchal fold in the fetuses (4.2 [2.5; 8.8] mm by ultrasound data). Prenatal screening was performed in the first trimester of pregnancy: from 11 weeks 0 days to 13 weeks 6 days. The study was carried out at the Genomed Ltd. (Moscow, Russia). The work was approved by the Ethics Committee of the Research Centre for Medical Genetics and conducted in accordance with the ethical standards set out in the Declaration of Helsinki (2013). Informed consent was obtained from all patients.

At the start, the fetal material was analyzed by standard karyotyping. When a normal karyotype was confirmed, the material was sent for CMA to test for submicroscopic chromosomal rearrangements.

Fetal DNA samples were examined using SNP-based CMA. The analysis was performed using a GeneChip™ Scanner 3000 system (Thermo Fisher Scientific, USA) containing 315,608 markers according to the manufacturer’s protocol. The CNVs were quantified by measuring the fluorescence intensity of the sample and reference DNA followed by calculating their ratio. The results were analyzed by the algorithm described in “Using CMA to improve the usefulness of medical genetic counseling” (developed at the Research Centre for Medical Genetics in 2015). The molecular karyotype was presented in accordance with the International System for Human Cytogenomic Nomenclature (ISCN 2016). After verification, all CNVs were analyzed for clinical significance. For this purpose, CNV characteristics such as size, localization, and gene content were compared with those described in the DGV, OMIM, ORPHANET, ISCA, and DECIPHER databases.

### Statistical processing

The qualitative indicators are presented as absolute and relative (%) values; the quantitative indicators are shown as the median and interquartile range. Statistical processing of the results was carried out using Microsoft Excel (2016) and Statistica 10.0 (StatSoft Inc., USA) programs. Statistical significance of the differences in values between the groups of patients without and with an increased NT was assessed using the c^2^ criterion and calculating the odds ratio (OR). Differences were considered statistically significant at p<0.05.

## Results

Invasive prenatal diagnostic procedures were performed in pregnant women at 12–17 weeks of gestation. Fetal material was obtained by amniocentesis in 149 women (66.2%), and by chorionic biopsy or placentocentesis — in 76 women (44.0%). Before the CMA step, all fetal samples underwent standard karyotyping, which revealed no deviations in the number and structure of the chromosomes.

The proportion of pathogenic CNVs was 9.8% (22/225). In 13 cases, deletions or duplications of one chromosome were detected; their spectrum is presented in Table 2.

**Table 2:** List of pathogenic CNVs identified in fetuses with normal karyotype

Genetic condition associated with identified CNVs	Molecular karyotype
2q33.1 deletion syndrome (ORPHA: 251028)	arr[hg19] 2q32.1q34(184355377_213928673)x1
8p23.1 deletion syndrome (ORPHA: 251071)	arr[hg19] 8p23.1(11547149_11701198)x1 arr[hg19] 8p23.1(8121171_12551618)x1
15q11.2 deletion syndrome (3 cases) (OMIM: 615656)	arr[hg19] 15q11.2(22770421_23713743)x1 (2 cases) arr[hg19] 15q11.2(22770421_23276605)x1
Mowat–Wilson syndrome (OMIM: 235730)	arr[hg19] 2q22.1(141142382_148859098)x1
16p13.11 duplication syndrome (ORPHA: 261243)	arr[hg19] 16p13.11(14866283_16328865)x3
Pathogenic duplication not classified as a syndrome	arr[hg19] 18p11.32(136226_14305057)x3 arr[hg19] 21q22.11(31873476_48097372)x3
Multiple imbalances of one chromosome	arr[hg19] 22q11.21(18644790_21798907)x1, 22q13.33(50861594_51197838)x3
Pathogenic triplication not classified as a syndrome	arr[hg19] 7q35q36.3(143217402_158947294)x4
Pathogenic deletion not classified as a syndrome	arr[hg19] 9q22.31(94729226_101015120)x1

In 9 cases, CNVs of two or more non-homologous chromosomes were identified. To determine the origin of the unbalanced translocations in fetal genomes, the parents’ karyotype data were retrieved. As a result, 8 out of 9 couples had balanced CA in one of the parents (Table 3); in one case, data on parents’ karyotype were not available.

**Table 3:** Karyotypes of parents who gave rise to fetuses with a chromosomal imbalance

No.	Fetus molecular karyotype	Parent karyotype
1	arr[hg19] 4q32.1q35.2(158117104_190957473)x1, 5p15.33p14.1(113576_26961133)x3	mother: 46,XX,t(4;5)(q31;p13)
		father: 46,XY
2	arr2q37.3(238807076_242783384)x1, 3q22.1(133552882_197851986)x3	mother: 46,XX
		father: 46,XY,t(2;3)(q37;q22)
3	arr1q43(238361421_249224684)x1, 5p15.33(113576_33967145)x3	mother: 46,XX
		father: 46,XY,t(1;5)(q43;p15)
4	arr4q32.1(157975815_190957473)x1, 5p15.33(113576_26961133)x3	mother: 46,XX,t(4;5)(q31;p13)
		father: 46,XY
5	arr[hg19] 2p25.3p13.2(12770_71782809)x3, 19p13.3p12(260911_23867121)x1[0.25], Xp22.33q26.3(2696761_136455694)x3	mother: 46,XX,t(X;2)(q26;p25)
		father: 46,XY
6	arr2q37.3(238092121_242782258)x1, 3q22.1(133662947_197572477)x3	mother: 46,XX
		father: 46,XY,t(2;3)(q37;q22)
7	arr17q24.3(67205557-81041823)x3, 22q13.2(44180131-51197766)x1	mother: 46,XX,add(22)(q13)
		father: 46,XY
8	arr4p16.3p15.1(68345_35545006)x3, 5p15.33p14.1(113576_27932273)x1	mother: 46,XX
		father: 46,XY
9	arr12p13.33(173786_37857751)x3, Xp22.31(6486489_8156174)x1	No data available

The parents in case 8 had normal karyotypes. However, considering the presence of an unbalanced translocation in the fetus, it was decided to conduct fluorescent *in situ* hybridization — the FISH diagnostic test. According to the results, the mother had a balanced translocation between the 4^th^ and 15^th^ chromosomes — t(4;5)(p15;p14), which could not be identified using a standard cytogenetic test due to the submicroscopic size of this chromosomal rearrangement.

In order to assess how much the isolated thickening of the nuchal fold in fetuses with a normal karyotype increases the risk of pathogenic CNVs, we examined a group of patients who were clinically comparable to the main group but had neither congenital malformations, no CA markers, and no increased NT. This group included 202 women who were referred for invasive prenatal diagnostic tests due to a high risk of CA (>1:100) revealed by the combined prenatal screening and in whom a normal fetal karyotype was determined by cytogenetic analysis. The CMA method was able to detect an abnormal molecular karyotype in 2 cases: in one case, a deletion in the short arm of the 4^th^ chromosome — arr4p16.3(1159060_1992826)x1 was detected, in the other, it was a deletion in the long arm of the 22^nd^ chromosome — arr22q11.21(18169796_21103320)x1.

Thus, in the absence of soft CA fetal markers and the presence of a normal karyotype (as per the standard cytogenetic test) pathogenic CNVs were detected in 2 out of 202 cases (0.9%) with the help of CMA. This value was significantly different from that in fetuses where an isolated increase in NT was found: c^2^=15.49; p<0.001; OR=10.85 [2.52; 46.70].

## Discussion

When analyzing the results, we noted that in 14 out of 22 cases (63.6%), the CNVs identified in this study were not classified as syndromes in the established databases. Of those 14 cases, 9 (64.2%) were represented by CNVs of two non-homologous chromosomes; in such cases, there is a high probability of carrying balanced translocations in the parents. Identifying genome modifications of that type is of high importance since balanced structural chromosomal rearrangements can lead to repeated miscarriages in the future or to the birth of babies with congenital malformations.

The SNP-based CMA assay involving low-density microarrays can be used to search for both aneuploidies and pathogenic CNVs in fetuses with increased NT diagnosed by ultrasound from 11 weeks to 13 weeks 6 days of gestation. In our study, the minimum CNV size detected in fetuses with an enlarged NT was 157 thousand bp; this size is significantly less than the standard cytogenetic test is able to identify. Thus, the use of CMA as a first-line diagnostic test in the presence of an increased fetal NT can improve the ability to detect chromosomal imbalances, including those caused by pathological meiotic segregation of the parental reciprocal translocation.

Isolated thickening of the nuchal fold in the fetus, as confirmed by ultrasound, increases the likelihood of detecting pathogenic CNVs by >10 times (OR=10.84) compared with the absence of these ultrasound markers. Therefore, in such a situation, we propose testing the fetal material using CMA even in the presence of normal karyotyping.

## Conclusion

An increased thickness of the fetal nuchal fold is an important marker of not only aneuploidies but also CNVs in the form of microdeletions and microduplications; the submicroscopic size of these chromosome rearrangements does not allow their identification by the standard karyotyping method. One of the important advantages of chromosomal microarray analysis is the ability to detect CNVs in non-homologous chromosomes; this type of CNVs is indicative of a balanced chromosomal abnormality in one of the parents and, therefore, it potentiates the risk of fetal karyotype abnormalities in subsequent pregnancies.
